# Factors associated with emergency room visits and hospitalisation amongst low-income public rental flat dwellers in Singapore

**DOI:** 10.1186/s12889-019-7009-5

**Published:** 2019-06-07

**Authors:** Liang En Wee, Lian Leng Low, Julian Thumboo, Angelique Chan, Kheng Hock Lee

**Affiliations:** 1 Department of Infectious Diseases, Singapore General Hospital, Singapore, Singapore; 2 Department of Family Medicine and Continuing Care, Singapore General Hospital, Singapore, Singapore; 3 Department of Rheumatology, Singapore General Hospital, Singapore, Singapore; 4 Health Services and Systems Research, Duke-NUS Graduate Medical School, Singapore, Singapore

**Keywords:** Hospitalisation, Emergency room visits, Low income, Singapore

## Abstract

**Background:**

In Singapore, a densely urbanised Asian society, more than 80% of the population stays in public housing estates and the majority (90%) own their own homes. For the needy who cannot afford home ownership, public rental flats are available. Staying in a public rental flat is associated with higher hospital readmission rates and poorer access to health services. We sought to examine sociodemographic factors associated with hospital admissions and emergency room visits amongst public rental flat residents.

**Methods:**

We surveyed all residents aged ≥60 years in a public rental housing precinct in central Singapore in 2016. Residents self-reported their number of emergency room visits, as well as hospitalisations, in the past 6 months. We obtained information on residents’ sociodemographic characteristics, medical, functional and social status via standardised questionnaires. We used chi-square to identify associations between emergency room visits/hospitalisations and sociodemographic characteristics, on univariate analysis; and logistic regression for multivariate analysis.

**Results:**

Of 1324 contactable residents, 928 participated in the survey, with a response rate of 70.1%. A total of 928 residents participated in our study, of which 59.5% were male (553/928) and 51.2% (476/928) were ≥ 70 years old. Around 9% (83/928) of residents had visited the emergency room in the last 6 months; while 10.5% (100/928) had been admitted to hospital in the past 6 months. On multivariable analysis, being religious (aOR = 0.43, 95%CI = 0.24–0.76) and having seen a primary care practitioner in the last 6 months (aOR = 0.46, 95%CI = 0.27–0.80) were independently associated with lower odds of emergency room visits, whereas loneliness (aOR = 1.96, 95%CI = 1.13–3.43), poorer coping (aOR = 1.72, 95%CI = 1.01–3.03) and better adherence (aOR = 2.23, 95%CI = 1.29–3.83) were independently associated with higher odds of emergency room visits. For hospitalisations, similarly poorer coping (aOR = 1.85, 95%CI = 1.12–3.07), better adherence (aOR = 1.69, 95%CI = 1.04–2.75) and poorer functional status (aOR = 1.85, 95%CI = 1.15–2.98) were all independently associated with higher odds of hospitalisations, whereas those who were religious (aOR = 0.62, 95%CI = 0.37–0.99) and those who were currently employed (aOR = 0.46, 95%CI = 0.37–0.99) had lower odds of being hospitalised.

**Conclusion:**

In this public rental flat population, functional status, coping and adherence, and having a religion were independently associated with emergency room visits and hospitalisation. Residents who had seen a primary care practitioner in the last 6 months had lower odds of visiting the emergency room.

## Background

Low socioeconomic status (SES) is a recognised risk factor for increased healthcare utilisation, including hospital admissions and emergency room visits [1–3]. While individual-levels of SES are associated with healthcare utilisation, staying in a deprived area can also drive increased healthcare utilisation [4, 5]. It is well known that characteristics of the neighbourhood have an impact on the health and well-being of residents residing in these communities [6, 7]. The majority of these studies, though, have been conducted in Western societies; only in recent years have there been studies from urban Asian societies that explore the link between neighbourhood characteristics and health [8].

Singapore is one such example of a rapidly urbanising multi-ethnic Asian society. Home ownership is a key local indicator of socioeconomic status (SES) in Singapore. The majority of Singaporeans (≥ 85%) [9, 10] stay in public housing and home ownership rates are high (87.2%) [9]. For the needy (< 5% of population) who cannot afford their own home, heavily subsidized public rental housing is available [11]. An unique characteristic is that in Singapore, public rental housing blocks are built within the same locations as owner-occupied public housing apartments [12]. Staying in a public rental flat in Singapore has been correlated with poorer measures of physical and mental health, amongst adult Singaporeans; even after controlling for individual SES (eg. individual employment status, education, being a recipient of financial aid) [13–19]. Residents of low-SES public rental flats had higher prevalence of poorer physical and mental health, poorer management of chronic disease, and reduced access to health services [13–19]. In terms of physical health, staying in a low-SES community was associated with poorer hypertension management [20], as well as higher rates of chronic pain [13]. For mental well-being, staying in a low-SES rental flat neighbourhood was associated with poorer cognitive function [14] and higher depression rates [15] among the elderly. Overall, residents in public rental flats had lower health-related quality of life (HRQoL) compared to their counterparts staying in owner-occupied housing [16]. This also had an impact on access to health services- residents in public rental flats had lower access to cardiovascular and cancer screening [17–19] and were less likely to seek treatment from medical professionals [21]. Studies demonstrate that healthcare utilisation is higher amongst residents of public rental flats, with higher readmission risk and emergency room visits compared to the general population [22]. However, there is a lack of literature identifying which segments of the rental flat population may be most at risk. Given that staying in public rental housing appears to be a risk marker of poorer health [23], we were interested in identifying factors associated with admission risk and increased utilisation of hospital services within this vulnerable population. As such, we investigated sociodemographic and medical factors associated with hospital admissions and emergency room visits amongst public rental flat residents in a rental flat precinct in Singapore.

## Methods

### Study population

We conducted a cross-sectional community health survey involving all residents aged ≥60 years in 12 public rental housing blocks in the Chin Swee, Jalan Kukoh, Jalan Minyak and York Hill precincts from 28th December 2016 – 5th March 2017. These precincts are served by the SingHealth Regional Health System and are within 1 km radius from the Singapore General Hospital campus. The survey captured comprehensive information on the social and medical needs of older adults who reside in public rental housing in these precincts. Public rental housing is an area-level indicator of low socio-economic status in Singapore [23] and people qualifying for this heavily government subsidized housing belonged to the financially needy class. For this surveyeligible participants were citizens or permanent residents, aged 60 years or older. Residents who scored 6 or less on the Abbreviated Mental Test (AMT) (used as a cutoff for identifying cognitive impairment in our local population) [24] and did not have a proxy to answer the survey were excluded. All eligible participants in the household could be recruited. The Singapore Ministry of Health initially provided 1817 unique addresses of older adults aged 60 years or older living within the study area; after excluding residents who were not eligible based on citizenship status and/or the presence of cognitive impairment precluding informed consent, a total of 1324 residents were potentially eligible for participation in this study.

### Study methodology

#### Baseline information

At baseline, information on residents’ sociodemographic characteristics, medical, functional and social status was collected via interviewer-administered standardized questionnaires. Major components of the questionnaire include socio-demographic attributes and family make-up; health status and physical disabilities/limitations; health behaviours; social network and social isolation; psychological health and stressors; income sources and perception of financial adequacy; health care utilization; medication adherence; and quality of life. Interviews were performed by trained survey staff in the subject’s home who underwent standardized training prior to study commencement. Comorbidity burden was measured using the Charlson’s Comorbidity Index (CCMI) [25]. Functional status in basic activities of daily living (bADL) was also measured using the Katz Index, while social isolation was measured using the Lubben’s Social Network Score-12 (LSNS 12) [26]. Self-rated health-related quality of life (HRQoL) was quantified using the EQ5D [16]. Depression was measured using the Patient Health Questionnaire-2 (PHQ-2) [27]. Loneliness was measured using the UCLA 3-item loneliness scale [28]. Residents were also asked if they had experienced at least one stressful life event in the past 1 year [29]. We used the Partners in Health (PIH) scale to assess self-management behaviour, a scale that was developed to assess the self-management knowledge and behaviour in people with chronic disease [30]. There were three components of this scale: coping, symptom management/adherence, and knowledge [31]. The median score on the coping subscale was 26.00 (interquartile ratio, IQR = 21–29); the median score on the symptom management/adherence subscale was 35 (IQR = 28–39), and the median score on the knowledge subscale was 19 (IQR = 15–21). As the results were skewed, we dichotomised the results into “better coping” vs. “poorer coping”, “better adherence” vs “poorer adherence” and “better knowledge” vs. “poorer knowledge”, using the median values as the cutoffs. Being a recipient of community services was defined as indicating “yes” to the receipt of any of the following services: daycare, home health services, home help services, caregiver support services, home modifications, befriender visits, escort services/transportation services. Participating in social activities was defined as indicating “yes” to participation in any of the following activities: grassroots activities, activities organised by the Senior Activity Centre, going out with family members/friends, attending a place of worship. Participating in fitness activities was defined as indicating “yes” to the following: going for a walk (for exercise purposes), or playing a game of sport/exercise. Data on healthcare utilisation was also collected (hospitalisations and visits at the emergency department within the last 6 months). Having at least one visit to a primary care practitioner for any chronic medical condition in the past 6-months was operationally defined as answering “yes” to the question: have you seen a doctor in a public outpatient primary care clinic (polyclinic) or a general practitioner (GP) in a private primary care clinic in the past 6 months, for any chronic medical condition? In Singapore, primary health care is provided by both private GPs as well as public polyclinics. Residents were also asked whether they had consults with traditional Chinese medicine practitioners or traditional healers in the past 6 months. Consults with traditional medicine practitioners were not classified as equivalent to visits with a primary care practitioner.

### Statistical analysis

Descriptive statistics were computed for the study population. We used chi-square to identify associations between emergency room visits/hospitalisations and sociodemographic characteristics, on univariate analysis. To identify associations between emergency room visits/hospitalisations and sociodemographic characteristics on multivariate analysis, we used a criterion of *p* < 0.1 on univariate analysis as a cutoff for initial entry into multivariate logistic regression models. We then removed variables that did not meet the cutoff for statistical significance in a stepwise fashion to generate the most parsimonious model, which was subsequently presented as the final model. All statistical analysis was performed using STATA (Version 22.0, USA) and statistical significance was set at *p* < 0.05.

### Ethics approval

This study was approved by SingHealth Centralized Institutional Review Board (CIRB 2016/2242). Informed written consent was sought, and participation was voluntary.

## Results

Of 1324 eligible residents, 928 participated in the survey, with a response rate of 70.1%. Around 9% (83/928) of residents had visited the emergency room in the last 6 months; while 10.5% (100/928) had been admitted to hospital in the past 6 months. Less than 5%(2.58%, 24/928) of residents had visited the emergency room more than once in the last 6 months (min-max = 1–16 visits), while 4.4% (41/928) of residents had been hospitalised more than once in the last six months (min-max = 1–10 hospitalisations). The sociodemographic characteristics of our population are provided in Table 1.

**Table 1:** Sociodemographic factors in a Singaporean public rental flat population (*N* = 928)

*Sociodemographic factors*	N, (%)
Age	
Age < 70 yrs	452/928 (48.7)
Age ≥ 70 yrs	476/928 (51.3)
Gender	
Male	553/928 (59.5)
Female	375/928 (40.5)
Ethnicity	
Non-Chinese	143/928 (15.4)
Chinese	785/928 (84.6)
Religion	
Atheist	209/928 (22.5)
Religious	719/928 (77.5)
Marital status	
Not married	638/928 (68.8)
Married	290/928 (31.2)
Educational status	
No formal education	460/928 (49.5)
Formal education	468/928 (50.5)
Employment	
Not working	605/928 (65.1)
Working	323/928 (34.9)
Principal source of income from financial aid	
No	715/928 (77.0)
Yes	213/928 (23.0)
Staying alone	
Staying with someone	577/928 (62.1)
Staying alone	351/928 (37.9)
Participating in social activities	
No	126/928 (13.5)
Yes	802/928 (86.5)
Participating in fitness activities	
No	284/928 (30.6)
Yes	644/928 (69.4)
Being a recipient of community services	
No	421/928 (45.3)
Yes	507/928 (54.6)
bADLs	
No limitations in bADLs	732/928 (78.8)
Some limitations in bADLs	196/928 (21.2)
iADLs	
No limitations in iADLs	617/928 (66.4)
Some limitations in iADLs	311/928 (33.6)
Charlson Comorbidity Index	
CCMI = 0	337/928 (36.3)
CCMI≥1	591/928 (63.7)
Self-rated health (HRQoL)	
Not in perfect health	509/928 (54.8)
In perfect health	419/928 (45.2)

Factors associated with emergency room visits and hospitalisations on univariate analysis are illustrated in Table 2. Overall, on univariate analysis using chi-square testing, being non-religious, being lonely, having sustained a fall in the past 1 year, functional limitations (in both basic and instrumental activities of daily living, ADLs), poor coping, better adherence, increased comorbidity burden, and poorer self-rated health were associated with higher odds of emergency room visits (*p* < 0.05), whereas having visited a primary care practitioner in the last 6 months for routine review of any chronic medical condition was associated with lower odds of emergency room visits. For hospitalisations, being unemployed, being a recipient of community services, having depressive symptoms, experiencing a stressful life event, having visual problems, having sustained a fall in the past year, having functional limitations, having poor coping, higher comorbidity burden, and poorer self-rated health were all associated with hospitalisations (*p* < 0.05).

**Table 2:** Sociodemographic factors associated on univariate analysis with 6-month emergency room visits and hospitalisation in a Singaporean public rental flat population (*N* = 928)

	Attended emergency room in past 6 months (n%)	Unadjusted odds ratio (95% CI)	*p*-value	Hospitalised in past 6 months (n%)	Unadjusted odds ratio (95% CI)	*p*-value
*Sociodemographic factors*						
Age						
Age < 70 yrs	37/452 (8.2)	1.00	0.490	40/452 (8.8)	1.00	0.072
Age ≥ 70 yrs	46/476 (9.7)	1.20 (0.76–1.88)		60/476 (12.6)	1.49 (0.97–2.26)	
Gender						
Male	47/553 (8.5)	1.00	0.560	55/553 (9.9)	1.00	0.333
Female	36/375 (9.6)	1.14 (0.73–1.80)		45/375 (12.0)	1.24 (0.81–1.88)	
Ethnicity						
Non-Chinese	15/143 (10.5)	1.00	0.523	15/143 (10.5)	1.00	1.00
Chinese	68/785 (8.7)	0.81 (0.45–1.46)		85/785 (10.8)	1.04 (0.58–1.85)	
Religion						
Atheist	28/209 (13.4)	1.00	0.013	28/209 (13.4)	1.00	0.165
Religious	55/719 (7.6)	0.54 (0.33–0.87)		72/7119 (10.0)	0.72 (0.45–1.15)	
Marital status						
Not married	55/638 (8.6)	1.00	0.621	70/638 (11.0)	1.00	0.820
Married	28/290 (9.7)	1.13 (0.70–1.83)		30/290 (10.3)	0.94 (0.60–1.47)	
Educational status						
No formal education	41/460 (8.9)	1.00	1.00	56/460 (12.2)	1.00	0.204
Formal education	42/468 (9.0)	1.01(0.64–1.58)		44/468 (9.4)	0.75 (0.49–1.14)	
Employment						
Not working	62/605 (10.2)	1.00	0.070	84/605 (13.9)	1.00	< 0.001
Working	21/323 (6.5)	0.61 (0.36–1.02)		16/323(5.0)	0.32 (0.19–0.56)	
Principal source of income from financial aid						
No	60/715 (8.4)	1.00	0.276	73/715 (10.2)	1.00	0.315
Yes	23/213 (10.8)	1.32 (0.80–2.19)		27/213 (12.7)	1.28 (0.80–2.04)	
***Social network***						
Staying alone						
Staying with someone	49/577 (8.5)	1.00	0.554	55/577(9.5)	1.00	0.127
Staying alone	34/351 (9.7)	1.16 (0.73–1.83)		45/351 (12.8)	1.40 (0.92–2.12)	
Participating in social activities						
No	14/126 (11.1)	1.00	0.400	17/126 (13.5)	1.00	0.282
Yes	69/802 (8.6)	0.75 (0.41–1.38)		83/802 (10.3)	0.74 (0.42–1.30)	
Participating in fitness activities						
No	24/284 (8.5)	1.00	0.803	32/284 (11.3)	1.00	0.732
Yes	59/644 (9.2)	1.09 (0.67–1.80)		68/644 (10.6)	0.93 (0.60–1.45)	
Being a recipient of community services						
No	32/421 (7.6)	1.00	0.205	33/421 (7.8)	1.00	0.010
Yes	51/507 (10.1)	1.36 (0.86–2.20)		67/507 (13.2)	1.79 (1.16–2.78)	
Lubben’s Social Network Score (LSNS 12)						
LSNS< 20	27/336 (8.0)	1.00	0.618	27/336(8.0)	1.00	0.127
LSNS≥20	45/494 (9.1)	1.15 (0.70–1.89)		57/494 (11.5)	1.49 (.92–2.41)	
Depression (PHQ-2)						
Not depressed (0–2)	68/821 (8.3)	1.00	0.065	79/821 (9.6)	1.00	0.021
Depressed [3–6]	12/82 (14.6)	1.89 (0.98–3.68)		15/82 (18.3)	2.10 (1.15–3.89)	
Loneliness (UCLA 3-item)						
Not lonely [1–3]	44/640 (6.9)	1.00	0.008	57/640 (8.9)	1.00	0.071
Lonely [4–9]	29/225 (12.9)	2.00 (1.22–3.29)		30/225 (13.3)	1.57 (0.98–2.52)	
Experienced at least 1 stressful life event in the past year						
No	8/47 (17.0)	1.00	0.061	10/47 (21.3)	1.00	0.027
Yes	75/881 (8.5)	0.45 (0.21–1.00)		90/881 (10.2)	0.42 (0.20–0.88)	
***Functional status***						
Visual problems						
No visual problems	66/756 (8.7)	1.00	0.657	72/756 (9.5)	1.00	0.014
Has visual problems	17/172 (9.9)	1.15 (0.65–2.00)		28/172 (16.2)	1.85 (1.15–2.96)	
Hearing problems						
No hearing problems	72/812 (8.9)	1.00	0.862	82/812 (10.1)	1.00	0.107
Has hearing problems	11/116 (9.5)	1.08 (0.55–2.10)		18/116(15.5)	1.64 (0.94–2.84)	
Falls						
No falls	50/688 (7.3)	1.00	0.004	60/688 (8.7)	1.00	0.001
At least 1 fall in past year	33/240 (13.8)	2.03 (1.28–3.24)		40/240 (16.7)	2.09 (1.36–3.22)	
bADLs						
No limitations in bADLs	54/732 (7.4)	1.00	0.003	63/732 (8.6)	1.00	< 0.001
Some limitations in bADLs	29/196 (14.8)	2.18 (1.34–3.53)		37/196 (18.9)	2.47 (1.59–3.84)	
iADLs						
No limitations in iADLs	42/617 (6.8)	1.00	0.002	43/617 (7.0)	1.00	< 0.001
Some limitations in iADLs	41/311 (13.2)	2.08 (1.32–3.27)		57/311 (18.3)	3.00 (1.96–4.57)	
***Self-management***						
Coping (PIH coping subscale)						
Poorer coping	51/384 (13.3)	1.00	0.016	61/384 (15.9)	1.00	0.005
Better coping	27/351 (7.7)	0.54 (0.33–0.89)		31/351 (8.8)	0.51 (0.32–0.81)	
Adherence (PIH adherence subscale)						
Poorer adherence	39 /424 (9.2)	1.00	0.031	48/424 (11.3)	1.00	0.090
Better adherence	42/289 (14.5)	1.68 (1.06–2.67)		46 /289 (15.9)	1.48 (0.96–2.29)	
Knowledge (PIH knowledge subscale)						
Poorer knowledge	48/392 (12.2)	1.00	0.345	59/392 (15.1)	1.00	0.192
Better knowledge	33 (9.8)	0.78 (0.49–1.24)		39/336 (11.6)	0.74 (0.48–1.14)	
***Medical status***						
Charlson Comorbidity Index						
CCMI = 0	12/337 (3.6)	1.00	< 0.001	15/337 (4.5)	1.00	< 0.001
CCMI≥1	71/591 (12.0)	3.70 (1.98–6.92)		85/591(14.4)	3.61 (2.05–6.35)	
Smoking status						
Not smoking	63/689 (9.1)	1.00	0.793	79/689 (11.5)	1.00	0.277
Currently smoking	20/239 (8.4)	0.91 (0.54–1.54)		21/239 (8.8)	0.74 (0.45–1.23)	
Self-rated health (HRQoL)						
Not in perfect health	57/509 (11.2)	1.00	0.008	75/509 (14.7)	1.00	< 0.001
In perfect health	26/419 (6.2)	0.53 (0.32–0.85)		25/419 (6.0)	0.36 (0.23–0.59)	
***Medical consultation***						
Seen a primary care practitioner in past 6 months						
No	28/210 (13.3)	1.00	0.019	30/210 (14.3)	1.00	0.075
Yes	54/698 (7.7)	0.55 (0.34–0.89)		68/698 (9.7)	0.65 (0.41–1.03)	
Seen a traditional medicine practitioner in past 6 months						
No	70/750 (9.3)	1.00	0.545	85/750 (11.3)	1.00	0.323
Yes	12/158 (7.6)	0.78 (0.42–1.51)		13/58 (8.2)	0.70 (0.38–1.29)	

The factors associated with emergency room visits and hospitalisations on multivariate analysis, using multivariable logistic regression, are illustrated in Table 3. Being religious (aOR = 0.43, 95%CI = 0.24–0.76) and having visited a primary care provider in the last 6 months for routine review (aOR = 0.46, 95%CI = 0.27–0.80) were independently associated with lower odds of emergency room visits, whereas loneliness (aOR = 1.96, 95%CI = 1.13–3.43), poorer coping (aOR = 1.72, 95%CI = 1.01–3.03) and better adherence (aOR = 2.23, 95%CI = 1.29–3.83) were independently associated with higher odds of emergency room visits. For hospitalisations, similarly poorer coping (aOR = 1.85, 95%CI = 1.12–3.07), better adherence (aOR = 1.69, 95%CI = 1.04–2.75) and poorer functional status (aOR = 1.85, 95%CI = 1.15–2.98) were all independently associated with higher odds of hospitalisations, whereas those who were religious (aOR = 0.62, 95%CI = 0.37–0.99) and those who were currently employed (aOR = 0.46, 95%CI = 0.37–0.99) had lower odds of being hospitalised.

**Table 3:** Sociodemographic factors associated on multivariate analysis with 6-month emergency room visits and hospitalisation in a Singaporean public rental flat population (*N* = 928)

Attended emergency room in past 6 months	Adjusted odds ratio (95% CI)^a,b^	*p*-value
Religious (vs. atheist)	0.43 (0.24–0.76)	0.004
Loneliness (vs not lonely)	1.96 (1.13–3.43)	0.017
Poorer coping (vs. better coping)	1.72 (1.01–3.03)	0.050
Better adherence (vs. poorer adherence)	2.23 (1.29–3.83)	0.004
Seen a primary care practitioner in the past 6 months (vs. no visit with a primary care practitioner in the past 6 months)	0.46 (0.27–0.80)	0.005
Hospitalised in past 6 months	Adjusted odds ratio (95% CI)^a,c^	*p*-value
Religious (vs. atheist)	0.62 (0.37–0.99)	0.050
Working (vs. not currently working)	0.46 (0.25–0.85)	0.011
Poorer coping (vs. better coping)	1.85 (1.12–3.07)	0.016
Better adherence (vs. poorer adherence)	1.69 (1.04–2.75)	0.034
Some limitation in iADLs (vs. no limitation in iADLs)	1.85 (1.15–2.98)	0.002

^a^The most parsimonious model was utilised in the multivariate logistic regression models presented. All variables presented were significant on multivariate analysis (*p* < 0.05)

^b^ Goodness-of-fit was assessed via calculating the R^2^; in the final multivariate model, R^2^ = 0.63

^c^Goodness-of-fit was assessed via calculating the R^2^; in the final multivariate model, R^2^ = 0.65

## Discussion

While staying in a public rental flat is a clear marker of poorer health in our local population, those who require admission to hospital, or visit the emergency department are likely to be most in need. In our study, around 9% (83/928) of patients had attended the emergency room in the last 6 months; while 10.5% (100/928) had been admitted to hospital in the past 6 months. These figures are comparable with the higher rates of hospitalisation and emergency room visits amongst rental flat residents, which has previously been noted in the literature. In our previous study comparing healthcare utilisation between residents in rental and owner-occupied housing, 15% of the rental flat population had been hospitalised ≥3 times in 1 year, whereas 13% had visited the emergency department ≥4 times in 1 year. This was compared against rates of 10 and 5%, respectively, in the general population [22]. In this study, functional limitation was associated with higher odds of hospitalisation. This was not surprising, as increased functional limitation makes care in the community more challenging [32, 33]. Being religious was independently associated with lower odds of emergency room visits, as well as hospitalisation, in our rental flat population. We hypothesise that having a religion may have helped low-income residents improve mental resilience and coping [34]; studies in the local setting found that elderly people of all religious affiliations reported less frequent treatment by healthcare professionals for mental health problems [35], which are tied closely to physical health and are prevalent in our rental flat population [14, 15]. An alternative explanation might be that residents who identified affiliation with a religion also had access to community services provided by religious organisations. In Singapore, the “landscape of help” encompasses state structures that provide basic housing, insurance and social security, as well as a “many helping hands” approach, in which voluntary welfare organisations provide services and the state acts as an enabler [36]. Perhaps services provided by these religious organisations, such as free clinics, meals delivery services and befriender services, help to meet the physical and psychological needs of rental flat dwellers and reduce the odds of healthcare utilisation. Poorer coping, but better adherence, was associated with higher odds of emergency room visits as well as hospitalisation. Perhaps in this low-income population, individuals who were more engaged and adherent with their treatment plan had higher odds of visiting the emergency department or being hospitalised because of a lack of engagement with primary care [21].

Interestingly, there were some differences between the factors associated with healthcare utilisation in this public rental flat population, when compared against the population at large. Loneliness in the rental flat population was associated with higher odds of emergency room attendance. While studies in Western societies do demonstrate an association between loneliness and increased physician visits [37, 38], our observation (i.e loneliness associated with higher odds of healthcare utilisation) runs counter to previous studies in our local population that demonstrated a significant association between loneliness and lower odds of healthcare utilisation in the general population [39]. Perhaps lonely residents in disadvantaged rental flat populations utilise healthcare more frequently because smaller social networks provide less reserves of support to fall back on in the event of illness, and hence these individuals are affected disproportionately. Having at least one visit to a primary care practitioner for any chronic medical condition in the past 6-months was associated with lower, not higher, odds of emergency room visits in our rental flat population. Conversely, studies of healthcare utilisation in the general Singaporean population indicated that past healthcare use (eg. specialist clinic visits) was a predictor of higher likelihood of healthcare utilisation [32]. Other studies in Western populations did not demonstrate a link between primary care access and emergency room attendance in deprived populations [40]. We hypothesise that in our rental flat population, emergency room attendance is seen as the “last resort”, with residents preferring to self-medicate at home and avoiding preventive care [21]. Perhaps the association between regular medical care and lower odds of emergency room visits could be attributable to higher participation in preventive care for residents accessing regular primary care [17], and a willingness to seek medical consultation earlier, rather than later, when ill. In our previous qualitative study of attitudes to primary care amongst rental flat residents, rental flat residents expressed a marked desire to seek medical consultation only for “major” illnesses, but not “minor” ones [21]. However, definitions of minor and major ailments varied greatly, and some defined minor ailments as those with mild/tolerable symptoms, and major ailments as those with symptoms greatly affecting their quality of life, without identifying that both major and minor ailments could form part of the same underlying disease process [21]. Low-income Singaporeans (including all residents of public rental flats) benefit from the Community Health Assist Scheme (CHAS), which subsidises care at private general practitioners within the community for chronic diseases, with the aim of encouraging Singaporeans to turn to their family physicians first, rather than going straight to hospitals [41]. Given that visiting a primary care provider in the past 6 monthsis associated with lower odds of healthcare utilisation amongst lower-income Singaporeans, usage of primary care amongst rental flat residents can be further incentivised.

The limitations of our study are as follows. Given the cross-sectional nature of our study, we can only identify associations, but not causation. Additionally, this study was carried out in a single site; we were unable to obtain a nationally representative sample of the rental flat population in Singapore because of logistical difficulties, as rental flats are scattered across the entire country in socially-integrated precincts. Therefore, the findings may not be fully representative of the rental flat population as a whole. However, we note that our study population is fairly similar in terms of sociodemographic makeup when compared against national data on low-income neighbourhoods [11].

## Conclusion

In conclusion, in this public rental flat population, impaired functional status, loneliness and poorer coping were independently associated with emergency room visits and hospitalisation. Residents who had visited a Western primary care practitioner at least once in the past 6 months for chronic medical conditions had lower odds of visiting theemergency room. More can be done to strengthen access amongst lower-income Singaporeans to primary care, so as to potentially reduce preventable emergency room visits and hospitalizations. s.

## Data Availability

The datasets used and/or analysed during the current study are available from the corresponding author on reasonable request.

## Abbreviations

AMT: Abbreviated Mental Test
bADL: Basic activities of daily living
CCMI: Charlson’s Comorbidity Index
CHAS: Community Health Assist Scheme
HRQoL: Health-related quality of life
IQR: Interquartile ratio
LSNS-12: Lubbens’ Social Network Score-12
OR: Odds ratio
PHQ-2: Patient Health Questionnaire-2
SES: Socioeconomic status

## References

[1] Krumholz H, Bernheim S (2014). Considering the role of socioeconomic status in hospital outcomes measures. Ann Intern Med.

[2] Filc D, Davidovich N, Novack L, Balicer R (2014). Is socioeconomic status associated with utilization of health care services in a single-payer universal health care system?. Int J Equity Health.

[3] Scantlebury R, Rowlands G, Durbaba S, Schofield P, Sidhu K, Ashworth M (2015). Socioeconomic deprivation and accident and emergency visits: cross-sectional analysis of general practices in England. Br J Gen Pract.

[4] Weisz D, Gusmano M, Wong G, Trombley J (2015). Emergency department use: a reflection of poor primary care access?. Am J Manag Care.

[5] Rudge G, Mohammed M, Fillingham S, Girling A, Sidhu K, Stevens A (2013). The combined influence of distance and neighbourhood deprivation on emergency department attendance in a large English population: a retrospective database study. PLoS One.

[6] Roux A (2001). Investigating neighborhood and area effects on health. Am J Public Health.

[7] Yen I, Michael Y, Perdue L (2009). Neighborhood environment in studies of health of older adults: a systematic review. Am J Prev Med.

[8] Loo B, Lam W, Mahendran R, Katagiri K (2017). How is the neighborhood environment related to the health of seniors living in Hong Kong, Singapore, and Tokyo? Some insights forPromoting aging in place. Ann Am Assoc Geogr.

[9] Department of Statistics Singapore. Home ownership rate of resident households 2015 [updated 2015. Available from: https://www.singstat.gov.sg/find-data/search-by-theme/households/households/visualising-data.

[10] Housing & Development Board Singapore. Public housing in Singapore: Residents' profile, housing satisfaction and preferences: HDB Sample Household Survey; 2013.

[11] Housing & Development Board Singapore. Rents & Deposits 2013 [updated 15 May 2013. Available from: https://www.hdb.gov.sg/cs/infoweb/residential/renting-a-flat/renting-from-hdb/public-rental-scheme/rents-and-deposits.

[12] Housing & Development Board Singapore (2012). Ethnic integration policy and Singapore permanent resident quota.

[13] Wee LE, Sin D, Cher WQ, Li ZC, Shibli S, Koh GCH (2016). Chronic pain in a low socioeconomic status population in Singapore: a cross-sectional study. Pain Med.

[14] Wee L, Yeo W, Yang G, Hannan N, Lim K, Chua C (2012). Individual and area level socioeconomic status and its association with cognitive function and cognitive impairment (Low MMSE) among community-dwelling elderly in Singapore. Dement Geriatr Cogn Dis Extra.

[15] Wee L, Yong Y, Chng M, Chew S, Cheng L, Chua Q (2014). Individual and area-level socioeconomic status and their association with depression amongst community-dwelling elderly in Singapore. Aging Ment Health.

[16] Wee L, Peter D, Sim A, Lee R, Tay S, Luo N, et al. Health related quality of life in a low-socioeconomic status public rental flat population in Singapore. Appl Res Qual Life. 2017.

[17] Wee LE, Cher WQ, Sin D, Li ZC, Koh GC (2016). Primary care characteristics and their association with health screening in a low-socioeconomic status public rental-flat population in Singapore- a mixed methods study. BMC Fam Pract.

[18] Wee L, Koh G (2012). Individual and neighborhood social factors of hypertension management in a low-socioeconomic status population: a community-based case-control study in Singapore. Hypertens Res.

[19] Wee L, Koh G, Chin R, Yeo W, Seow B, Chua D (2012). Socioeconomic factors affecting colorectal, breast and cervical cancer screening in an Asian urban low-income setting at baseline and post-intervention. Prev Med.

[20] Wee L, Wong J, Chin R, Lin Z, Goh D, Vijakumar K (2013). Hypertension management and lifestyle changes following screening for hypertension in an Asian low socioeconomic status community: a prospective study. Ann Acad Med Singap.

[21] Wee L, Lim L, Shen T, Lee E, Chia Y, Tan A (2014). Choice of primary health care source in an urbanized low-income community in Singapore: a mixed-methods study. Fam Pract.

[22] Low L, Wah W, Ng M, Tan S, Liu N, Lee K (2016). Housing as a social determinant of health in Singapore and its association with readmission risk and increased utilization of hospital services. Front Public Health.

[23] Chan C, Lee K, Low L (2018). A systematic review of health status, health seeking behaviour and healthcare utilisation of low socioeconomic status populations in urban Singapore. Int J Equity Health.

[24] Sahadevan S, Lim PP, Tan NJ, Chan SP (2000). Diagnostic performance of two mental status tests in the older chinese: influence of education and age on cut-off values. Int J Geriatr Psychiatry.

[25] Charlson M, Pompei P, Ales K, Mackenzie C (1987). A new method of classifying prognostic comorbidity in longitudinal studies: development and validation. J Chronic Dis.

[26] Leung Y, Teo S, Chua M, Raman P, Liu C, Chan A (2016). Living arrangements, social networks and onset or progression of pain among older adults in Singapore. Geriatr Gerontol Int.

[27] Kroenke K, Spitzer R, Williams J (2003). The patient health Questionnaire-2: validity of a two-item depression screener. Med Care.

[28] Hughes M, Waite L, Hawkley L, Cacioppo J (2004). A short scale for measuring loneliness in large surveys. Res Aging.

[29] Lim M, Lim D, Gwee X, Nyunt M, Kumar R, Ng T (2015). Resilience, stressful life events, and depressive symptomatology among older Chinese adults. Aging Ment Health.

[30] Veldman K, Reijneveld S, Lahr M, Uittenbroek R, Wynia K (2017). The Partners in Health scale for older adults: design and examination of its psychometric properties in a Dutch population of older adults. Health Expect.

[31] Chiu T, Tam K, Siu C, Chau P, Battersby M (2017). Validation study of a Chinese version of Partners in Health in Hong Kong (C-PIH HK). Qual Life Res.

[32] Low L, Liu N, Wang S, Thumboo J, Ong M, Lee K (2016). Predicting frequent hospital admission risk in Singapore: a retrospective cohort study to investigate the impact of comorbidities, acute illness burden and social determinants of health. BMJ Open.

[33] Paul P, Heng B, Seow E, Molina J, Tay S (2010). Predictors of frequent attenders of emergency department at an acute general hospital in Singapore. Emerg Med J.

[34] Xu J (2018). Buddhism-as-a-meaning-system for coping with late-life stress: a conceptual framework. Aging Ment Health.

[35] Ng T, Nyunt M, Chiam P, Kua E (2011). Religion, health beliefs and the use of mental health services by the elderly. Aging Ment Health.

[36] Mathi B, Mohamed S. Unmet social needs in Singapore: Singapore’s social structures and policies, and their impact on six vulnerable communities. In: Innovation LCfS, editor. Social insight research series. Singapore.2011.

[37] Gerst-Emerson K, Jayawardhana J (2015). Loneliness as a public health issue: the impact of loneliness on health care utilization among older adults. Am J Public Health.

[38] Taube E, Kristensson J, Sandberg M, Midlöv P, Jakobsson U (2015). Loneliness and health care consumption among older people. Scand J Caring Sci.

[39] Lim K, Chan A (2017). Association of loneliness and healthcare utilization among older adults in Singapore. Geriatr Gerontol Int.

[40] Harris M, Patel B, Bowen S (2011). Primary care access and its relationship with emergency department utilisation: an observational, cross-sectional, ecological study. Br J Gen Pract.

[41] Ng S. 5 things about the community health assist scheme or CHAS. The Straits Times. 2014 May 17th, 2014.

